# Bioinspired Intelligent Algorithm and Its Applications for Mobile Robot Control: A Survey

**DOI:** 10.1155/2016/3810903

**Published:** 2015-12-27

**Authors:** Jianjun Ni, Liuying Wu, Xinnan Fan, Simon X. Yang

**Affiliations:** ^1^College of IOT Engineering, Hohai University, Changzhou 213022, China; ^2^Changzhou Key Laboratory of Special Robot and Intelligent Technology, Hohai University, Changzhou 213022, China; ^3^Advanced Robotics and Intelligent Systems (ARIS) Laboratory, School of Engineering, University of Guelph, Guelph, ON, Canada N1G 2W1

## Abstract

Bioinspired intelligent algorithm (BIA) is a kind of intelligent computing method, which is with a more lifelike biological working mechanism than other types. BIAs have made significant progress in both understanding of the neuroscience and biological systems and applying to various fields. Mobile robot control is one of the main application fields of BIAs which has attracted more and more attention, because mobile robots can be used widely and general artificial intelligent algorithms meet a development bottleneck in this field, such as complex computing and the dependence on high-precision sensors. This paper presents a survey of recent research in BIAs, which focuses on the research in the realization of various BIAs based on different working mechanisms and the applications for mobile robot control, to help in understanding BIAs comprehensively and clearly. The survey has four primary parts: a classification of BIAs from the biomimetic mechanism, a summary of several typical BIAs from different levels, an overview of current applications of BIAs in mobile robot control, and a description of some possible future directions for research.

## 1. Introduction

Recently, a new type of intelligent computing methods has been developed to overcome the limitations of traditional artificial intelligent methods. One of the most important features of these intelligent computing methods is that their working mechanisms are more lifelike to an individual or a group of organisms, which can be understood very well. And these methods usually have higher efficiency than the traditional artificial intelligent methods. The intelligent computing methods of this type are defined as bioinspired intelligent algorithms (BIAs) to distinguish them from the traditional artificial intelligent methods. BIAs have made significant progress in both understanding of the neuroscience and biological systems and applying to various fields, such as mobile robot control.

The purpose of this paper is to provide a survey of the recent research in the bioinspired intelligent algorithm and its applications for mobile robot control. We focus on the research in the realization of various BIAs based on different mechanisms. To help in understanding BIAs, we focus exclusively on the applications for mobile robot control that is a main application field of BIAs. Several other surveys of the literature in the field of BIAs and mobile robot control are available that complement this paper (see, e.g., [1, 2]).

The main contributions of this paper are summarized as follows. (1) A detailed analysis on the features of BIAs is given out. And a new classification is proposed from the biomimetic mechanism of BIAs. (2) A survey of recent BIAs is provided, and some BIAs are chosen elaborately, to focus on the biomimetic mechanism and realization processing of BIAs. (3) An overview of the applications in mobile robot control by BIAs is given out, to further illustrate BIAs. Furthermore, some possible future directions of the research on BIAs are discussed.

This paper is organized as follows: In Section 2, we provide a general introduction of BIAs and give out a classification of these BIAs.  Section 3 analyzes and summarizes some typical BIAs. The main applications of BIAs in mobile robot control are introduced in Section 4.  Section 5 discusses the future research directions for the theory of BIAs and their applications in mobile robot control. Finally, conclusions are given out in Section 6.

## 2. Classification of BIAs

There are two main aspects for human beings to learn from nature. The first one is to make inventions by imitating the structures of organisms. For example, human beings created the manned glider flight based on the flying principle of birds and invented the echolocation sonar system inspired from the echolocation function of bats. Another one is to design some algorithms (technologies) inspired from various principles of nature. For example, based on the process of ant foraging, Ant Colony Algorithm was developed; and based on the process of natural evolution, such as inheritance, mutation, selection, and crossover, Genetic Algorithm was proposed. With the development of science and technology, human beings will face more and more challenges. Some of the traditional artificial intelligent algorithms are too simple in structures or functions to satisfy the requirements of developments. From the late 20th century, more and more novel artificial intelligent algorithms have been proposed, such as bioinspired neural network, Artificial Immune Algorithm, and Membrane Computing Algorithm. These novel artificial intelligent methods as well as some other intelligent algorithms (such as Ant Colony Algorithm and Genetic Algorithm mentioned above) have a common feature. Namely, they have more lifelike biological working mechanisms than other types. The artificial intelligent methods of this type are the bioinspired intelligent algorithms (BIAs) discussed in this paper.

Now, BIAs are still in the stage of development, so there is no strict definition and uniform classification. Binitha and Sathya [1] described the origin and advantages of the bioinspired computing algorithms and pointed out that BIAs were heuristic methods that imitated the strategy of nature, which was a simple and nonrepresentational definition of BIAs. Bongard [3] introduced the development process of the bioinspired computing algorithm and analyzed the relationship between the BIAs and the traditional intelligent computing methods. Based on our expertise, the bioinspired intelligent computing algorithm can be defined as follows: it is a type of intelligent computing methods with a quite lifelike biological working mechanism, to imitate the function and structure of the organism, the individual and swarm behaviors, and the evolution process of life and society. However, it is not an easy job to exclude those methods from BIAs, which are not strictly bioinspired. In this paper, we do not intend to distinguish different types of intelligent computing algorithms but to analyze the main features of BIAs, classify these algorithms from the simulated biological working mechanisms, and survey different categories focused on the realization processing as well as the applications for mobile robot control.

Based on our research work and the overview of lots of literature, the remarkable features of BIAs discussed in this paper are summarized as follows [1, 4–7]:

1. Bioinspired feature: the working mechanism is very close to the biological or ecological mechanism of natural organisms. The bioinspired algorithms imitate the biological nature as much as possible to deal with the real-world problems.
2. Simplicity and emergence: the strategy and computation are often very simple, but their resultant effects are very amazing, which reflects the principle of emergence.
3. Robustness: these algorithms have strong robustness against the change of environments, parameters, and tasks; namely, these algorithms have good applicability and flexibility.
4. Self-organization: these algorithms can improve the adaptability by self-learning or self-organization and realize the evolution successfully.
5. Other features: these algorithms have some other good features, such as parallelism in essence and nondeterminism.

**Figure 1:**
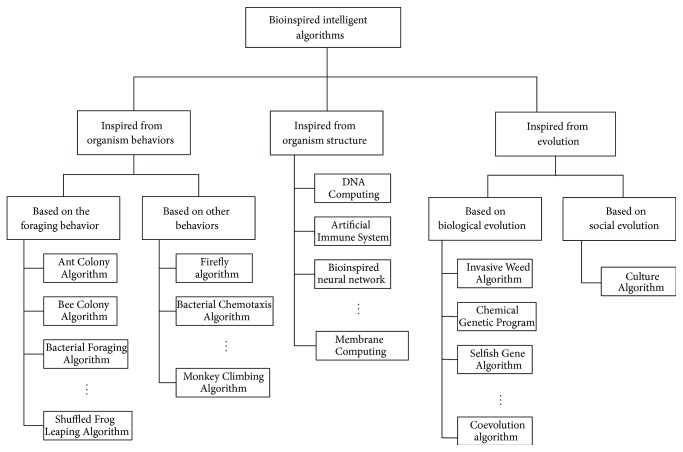
The classification of BIAs from the biomimetic mechanism.

As introduced above, BIAs have many excellent characteristics that can meet the needs of researchers. To introduce BIAs clearly and understand them easily, BIAs should be classified. From different view angle, various classifications can be obtained; for example, from the whole effects, we can see all BIAs as a type of evolutional optimization algorithms. In this paper, we will classify BIAs from their sources of the biomimetic mechanism; then BIAs can be divided into three categories: (1) inspired from organism behaviors; (2) inspired from organism structure; (3) inspired from evolution. The classification in this paper is single; that is to say, one bioinspired algorithm will not be classified into two or more different categories. The classification map is shown in Figure 1, and each category will be introduced in the next section clearly by some typical algorithms.

## 3. Theories Overview

There are some similarities in the property and application of each category with the same biomimetic mechanism. In this paper, we will focus on the realization processing of several novel algorithms proposed in the last two decades, as well as some distinctive or representative methods as far as we know.

### 3.1. Inspired from Organism Behaviors

Every living creature can survive and protect itself in its own way, such as foraging of food, defending against natural enemy, and courtship displaying. Based on the characteristics of these important behaviors of organisms, some BIAs have been developed. In these BIAs, most of them are inspired from the foraging behaviors of organisms, such as Ant Colony Algorithm, Bee Colony Algorithm, Bacterial Foraging Algorithm, Fish Swarm Algorithm, and Shuffled Frog Leaping Algorithm. There are also some algorithms which are inspired from other behaviors of organisms. For example, Monkey Climbing Algorithm simulates the climbing behavior of monkeys, and Bacterial Chemotaxis Algorithm simulates the chemotaxis behavior of bacteria. There are several other surveys of the literature on the behavior-based BIAs (see [8–10]), so we just select two algorithms to introduce the realization process in detail of the foraging behavior- (Bacterial Foraging Algorithm) and other behaviors- (Monkey Climbing Algorithm) based BIAs, respectively.

#### 3.1.1. Bacterial Foraging Algorithm

**Figure 2:**
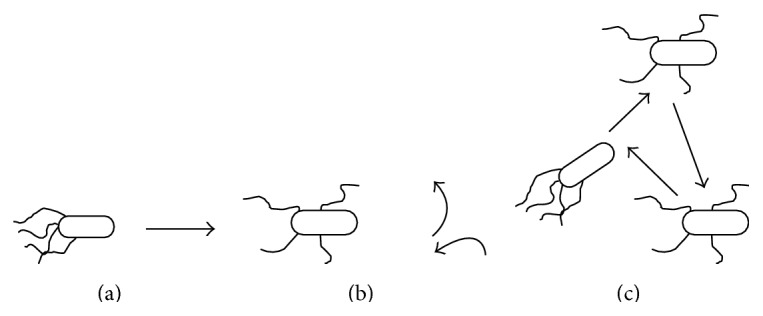
Swimming, tumbling, and chemotactic behavior of* E. coli* [11]: (a) the behavior of swimming; (b) the behavior of tumbling; (c) the behavior of chemotaxis.

In 2002, Passino proposed a novel BIA inspired from the behavior of* E. coli* bacterium of foraging nutrition and food in people's intestinal canal, which is called Bacterial Foraging Algorithm [11]. There are some special behavior patterns in bacterial foraging, such as swimming, tumbling, and chemotactic behavior (see Figure 2). The basic steps of Bacterial Foraging Algorithm are abstracted from these behaviors, which are introduced as follows [11–13].

*(1) Chemotaxis.* This is the behavior of bacteria gathering into the nutrition area, which includes tumbling and swimming. Swimming means moving to an arbitrary direction in a unit step, and tumbling decides the directions of the bacteria. Bacterium will refresh its fitness value at each step of tumbling and will not stop tumbling till the fitness value is no longer better or the number of steps is out of limitation. Let *θ* ^*i*^(*j*, *k*, *l*) represent the state of the *i*th bacterium (the location of the *i*th bacterium); then the tumbling behavior can be denoted by

(1) $$\begin{array}{c} \theta^{i}\left(\;j+1,k,l\;\right)=\theta^{i}\left(\;j,k,l\;\right)+C\left(\;i\;\right)\frac{\Delta \left(i\right)}{\sqrt{\Delta^{T}\left(i\right)\Delta \left(i\right)}}, \end{array}$$

where *i* is the sequence number of the bacterium; *k* is the number of reproduction; *j* is the number of chemotaxis; *l* is the number of dispersal; *C*(*i*) denotes a basic chemotactic step size; *θ* ^*i*^(*j* + 1, *k*, *l*) represents the next state of the *i*th bacterium after tumbling; Δ(*i*) is a random vector whose elements are random numbers in [−1,1]. The swimming behavior can be denoted by

(2) $$\begin{array}{c} \theta^{i}\left(\;j+1,k,l\;\right)=\theta^{i}\left(\;j+1,k,l\;\right)+C\left(\;i\;\right)\frac{\Delta \left(i\right)}{\sqrt{\Delta^{T}\left(i\right)\Delta \left(i\right)}}. \end{array}$$

*(2) Reproduction.* This is the behavior of selective multiplication of the bacteria. Namely, the bacteria with good fitness are selected to propagate, whose offsprings will have the same positions and steps. But the bacteria with bad fitness will be weeded out. The elimination mechanism is based on the sum of the costs of the bacterium when it goes over its lifetime. The health of the *i*th bacterium *J*_health_^*i*^ is calculated by

(3) $$\begin{array}{c} J_{health}^{i}=\overset{N_{c}+1}{\underset{j=1}{\sum }}J\left(\;i,j,k,l\;\right), \end{array}$$

where *J*(*i*, *j*, *k*, *l*) is the cost at the location of the *i*th bacterium; *N*_*c*_ is the length of the lifetime of the *i*th bacterium as measured by the number of chemotactic steps. The higher cost means the lower health, so the bacteria with the highest values of *J*_health_ will die at first.

*(3) Dispersal.* This is the behavior of dispersing the bacteria to random positions at a certain probability. This behavior aims at avoiding the local minimum of Bacterial Foraging Algorithm and realizing global search.

There are many advantages in Bacterial Foraging Algorithm, such as parameter insensitivity, strong robustness, and easy implementation. And a lot of research results have been obtained by this algorithm; for example, Sanyal et al. [14] presented an adaptive Bacterial Foraging Algorithm for the segmentation of gray images, where the Bacterial Foraging Algorithm is employed for maximization of fuzzy entropy to achieve the desired threshold based segmentation; Liu et al. [15] proposed a mobile robot path planning method based on Bacterial Foraging Algorithm, where the Bacterial Foraging Algorithm is used to minimize the path length and the number of turns without colliding with an obstacle.

#### 3.1.2. Monkey Climbing Algorithm

In 2008, Zhao and Tang [16] presented Monkey Climbing Algorithm inspired by the actions of monkeys when they climb the trees to the best place for some reasons, to solve global optimization problems. There are three main processes in this algorithm, namely, climb process, watch-jump process, and somersault process. (1) Climb process: this process represents the local search of the algorithm. The monkeys climb from the initial positions and find the respective local optimum positions. (2) Watch-jump process: this process represents the search process from local optimum to global optimum. The monkeys will look around when arriving at the local optimal positions. If some better positions can be found, the monkeys will jump to these new positions; otherwise, they will stay where they are. (3) Somersault process: this process represents the search process for avoiding the local optimum. The monkeys will somersault to new positions at a certain probability and begin a new search.

The numerical computing processes of Monkey Climbing Algorithm are summarized as follows [16, 17].

**Step 1 .**

Initialize the size of monkeys *M* and the initial positions of all the monkeys. For the *i*th monkey, its position is denoted by *x*_*i*_ = (*x*_*i*1_, *x*_*i*2_,…, *x*_*in*_), where *n* is the dimension of the solution.

**Step 2 .**

Optimize the positions of the monkeys by climbing. Set *y* = (*y*_1_, *y*_2_,…, *y*_*n*_), and *y*_*i*_ = *x*_*ij*_ + *a* · sgn(*f*_*ij*_′(*x*_*i*_)), where sgn(·) is a sign function; *a* > 0 is the step length of the climb process; and *f*_*ij*_′(*x*_*i*_) is the pseudo gradient of the objective function *f*(·) at the position *x*_*i*_, which is calculated by

(4) fij′xi=fxi+Δxi−fxi−Δxi2Δxij,j=1,2,…,n,

where Δ*x*_*i*_ = (Δ*x*_*i*1_, Δ*x*_*i*2_,…, Δ*x*_*in*_) and Δ*x*_*ij*_ = *a* or −*a* with the same probability. If position *y* is feasible, update the position of the monkey *x*_*i*_ with *y*.

**Step 3 .**

Search for better places within the visual range of monkeys and update their positions. The detailed computing process is as follows: generate a number *y*_*j*_⊆(*x*_*ij*_ − *b*, *x*_*ij*_ + *b*), where *b* is a constant to denote the eyesight of the monkey. If *f*(*y*) ≥ *f*(*x*_*i*_) and position *y* is feasible, the *i*th monkey will move from positions *x*_*i*_ to *y*, and go to Step 2.

**Step 4 .**

Generate a random number *α* ∈ [*c*, *d*], where [*c*, *d*] is the somersault interval. Get a new *y*_*j*_ = *x*_*ij*_ + *α*(*p*_*j*_ − *x*_*ij*_), where *p*_*j*_ = (1/*M*)∑_*i*=1_^*M*^ *x*_*ij*_,  *j* = 1,2,…, *n*. If position *y* is feasible, then force the monkey to go to the new position *y* and begin a search again.

**Step 5 .**

If the results meet the end conditions, the search process is ended and output the position vector (the optimal solution). Otherwise, go to Step 2.

**Remark 1 .**

Every step in this algorithm (including Steps 2, 3, and 4) will be repeated if the feasible and optimal positions cannot be found, till the number of iteration of each step meets the maximum or some conditions are satisfied.

One advantage of Monkey Climbing Algorithm is the pseudo-gradient calculation of the objective function in the climb process, which only requires two measurements of the objective function regardless of the dimension of the real-world problem. This advantage can reduce the cost of calculation and avoid the local minimum problem. In addition, Monkey Climbing Algorithm has a few parameters to adjust, which makes it particularly easy to implement. For example, Yi et al. [18] used a collaborative Monkey Climbing Algorithm to deal with the optimal sensor placement problem for health monitoring of high-rise structure. The effectiveness of the proposed algorithm is demonstrated by a numerical example with a high-rise structure, and the results show that the proposed algorithm can provide a robust design for sensor networks. Suguna and Maheswari [19] proposed a monkey search optimization based feature selection and extraction approach for performing image classification and recognition in the medical image segmentation process. The results show that the proposed approach is an appropriate algorithm for image segmentation.

### 3.2. Inspired from Organism Structure

Nature creates all things, where life is the most amazing one. Each part of life composition can give us some inspirations. For example, inspired from the double helix structure and complementary base pairing rule of DNA, Adleman proposed DNA Computing [20]; inspired from the structure of biological cells and the cooperation among different cells, Paun presented the concept of Membrane Computing [21]; inspired from the immune mechanism of biological immune system, Bersini and Varela firstly proposed an Artificial Immune System based method to deal with real-world problems [22]. Furthermore, inspired from the linked structure and the information transmission mechanism in human beings or animal brain and neuron system, some bioinspired neuron networks are proposed [23, 24].

In this section, three typical BIAs inspired from different levels of life are given out in detail, respectively, namely, DNA Computing (the level of chromosome), Membrane Computing (the level of cell), and Artificial Immune System (the level of system).

#### 3.2.1. DNA Computing

The study results of modern molecular biology indicate that the very complex structure of living body is the operation results on genetic information represented by DNA sequence. As “00” and “10” are used to represent information in computer, a single strand of DNA can be looked at as a method for representing decoding information in the multituple Σ = {A, G, C, T}, where A, G, C, and T denote adenine, guanine, cytosine, and thymine, respectively. Then the biological enzymes and other biochemical operations are the operators acted on the DNA sequence. Based on these ideas above, Adleman published the paper titled “Molecular Computation of Solutions to Combinatorial Problems” in the journal Science (1994), which means the birth of a new research field: DNA Computing [25].

**Figure 3:**
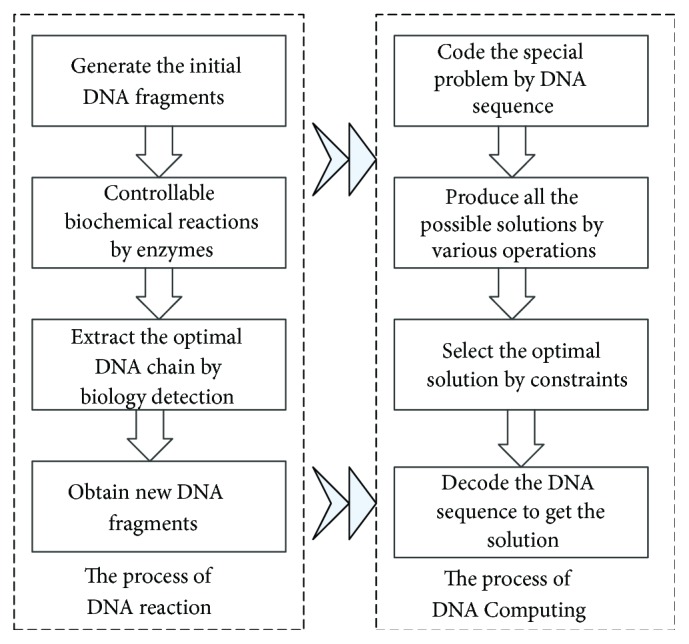
The mapping between the DNA reaction in biology and the process of DNA Computing.

The mapping between the DNA reaction in biology and the process of DNA Computing is shown in Figure 3. The basic process of DNA Computing is (1) to code a problem, by using the special double helix structure and complementary base pairing rules of DNA, and to map objects into DNA molecular chain; (2) to produce various data pools by the actions of biological enzymes in the test tube with DNA solution; (3) to map the data operations of the original problem into the controllable biochemical processes according to certain rules; (4) to obtain the calculating results by using molecular biological techniques, such as polymerase chain reaction, parallel overlap assembly, affinity chromatography, molecular purification, and magnetic bead separation.

DNA Computing has two major advantages by using the features of biological molecule and its biochemical reactions that are the massive storage and the parallel capacity. It has been applied in various fields and played a huge role, such as classification, information security, and robotic control [26–28]. Of course, there are still some disadvantages needed to be further studied in DNA Computing. For example, the information of DNA cannot be copied and is difficult to transplant.

#### 3.2.2. Membrane Computing

Membrane Computing was firstly presented by Paun in a research report in 1998, and the related paper was published in 2000 [29]. The goal of Membrane Computing is not intended to model the function of biological membranes but to get some new computing ideas and design new computing models, inspired from the cooperation among living cells and the cells of tissues, organs, or other structures. In general, a membrane system (also called P-system; see [29] for details) with *m* degree can be denoted by a multicomponent system:

(5) $$\begin{array}{c} \Pi =\left(\;V,T,C,\mu ,w_{1},\ldots ,w_{m},\left(\;R_{1},\rho_{1}\;\right),\ldots ,\left(\;R_{m},\rho_{m}\;\right)\;\right), \end{array}$$

where *V* is an alphabet, whose elements are called objects; *T*⊆*V* is an output alphabet; *C*⊆*V* − *T* is an activator; *μ* is a membrane structure containing *m* membranes; *w*_*i*_ ∈ *V* ^*∗*^ is a multiset of the objects associated with the region *i* of *μ*, where *V* ^*∗*^ is a set of arbitrary strings composed of the strings in *V*; *R*_*i*_ is a finite set of evolution rules; and *ρ*_*i*_ is a partial order relation over *R*_*i*_, which is called a priority relation on the rule of *R*_*i*_. The evolution rule can be denoted by a pair (*u*, *v*), where *u* is a string in *V*, *v* = *v*′ or *v* = *v*′*δ* (*v*′ is a string over the set {*a*_here_, *a*_out_, *a*_in_*j*__∣*a* ∈ *V*,  1 ≤ *j* ≤ *m*}, where *a*_here_, *a*_out_, and *a*_in_*j*__ are objects with different region destinations, and *δ* is a special symbol not in *V*). If a rule containing *δ* is performed, this membrane is dissolved.

In a nutshell, the membrane system is constructed of three parts: hierarchy of membranes, multiple sets for objects, and evolutionary rules. The membrane system uses evolutionary rules in the membrane structure to complete the computing, starting from the initial state (represented by multiple sets of objects) [21, 30].

The membrane system is a distributed, massively parallel, and nondeterministic computational model. Since the emergence of Membrane Computing, it has attracted a lot of attention and become a powerful theoretical framework in the field of natural computing. For example, Cheng et al. [31] presented a membrane algorithm for numerical optimization, which is an appropriate combination of a differential evolution algorithm, a local search, and P-systems. The effectiveness of the algorithm was tested on extensive numerical optimization experiments, and the results show that this algorithm performs better than its counterpart differential evolution algorithm. Zhang et al. [32] presented a membrane algorithm based on PSO for solving broadcasting problems, which are regarded as NP-hard combinatorial optimization problems. The experimental results from various broadcasting problems show that the proposed algorithm has better search capability, efficiency, solution stability, and precision than other optimization methods reported in the literature. In addition, Membrane Computing can be used in many other fields, such as computer graphics, linguistics, economics, computer science, and cryptography [30, 33, 34].

#### 3.2.3. Artificial Immune System

Biological immune system is a complex adaptive system in nature. The purpose of the immune system is to identify all cells (or molecules) in the body and to distinguish the self and nonself. Then the nonself cells are further classified in order to construct an appropriate defense mechanism. Artificial Immune System is a bioinspired intelligent computing method inspired by the principles and processes of the vertebrate immune system. With the advances in biology, more and more immune mechanisms are applied in computing algorithms including B-cells, T-cells, antibody, antigen, immunological learning, immunological memory, clone selection, and immunological response.

At present, the theoretical study of Artificial Immune System focuses on four aspects. (1) Artificial immune network model: currently two influential artificial immune network models are Resource-Limited Artificial Immune System and aiNet [35]. (2) Clone selection: clone selection is an important doctrine of biological immune system theory, and its basic idea is that only those cells who can recognize antigen will amplify to be selected and retained [36]. (3) Immune evolutionary algorithm: immune evolutionary algorithm is a general name of various immune optimization algorithms, which is developed from an evolutionary algorithm framework by introducing many features of the immune system [5]. (4) Negative selection algorithm: negative selection is an important mechanism to make the immune system have the function of immune tolerance. The process is that the immature T-cells will die if they reply to self during the growth in the thymus. The mature T-cells will be distributed in the circulatory system of human body, combine with the nonself, and tolerate self. Based on this self-nonself principle of the immune system, Forrest et al. [37] proposed the negative selection algorithm, which has become one of the main artificial immune methods for their unique characteristics.

Artificial Immune System has some outstanding qualities, such as noise patience, learning without teacher, self-organization, and no need of negative example, so it has been used widely. For example, Li et al. [35] proposed an artificial immune network based anticollision algorithm for dense RFID readers, where the major immune operators are designed to satisfy the practical convention of RFID systems, and the experiments results show that the artificial immune network based method is effective and efficient in mitigating the reader-to-reader collision problem in dense RFID networks. Cai et al. [38] introduced the clonal selection algorithm into the problem of the recognition of community detection in complex networks, and the experiments on both synthetic and real-world networks demonstrate the effectiveness of the proposed method. Artificial Immune System is suited for dealing with the problems of feature selection, anomaly detection, and so on [39–41].

### 3.3. Inspired from Evolution

Natural selection and survival of the fittest is the world's eternal unchanging law. In biology, the evolution refers to the changes in the population genetic traits between generations. Natural selection can favor the genetic traits of survival and reproduction to become more common and the harmful traits to become rarer. Human beings as advanced living creatures form a special social system which evolves mainly on social culture. Inspired from these complex evolution processes of lives, some evolution algorithms are proposed, such as Genetic Algorithm, Evolutionary Strategy, Chemical Genetic Program, Invasive Weed Algorithm, and Culture Algorithm.

From three different evolution levels, three BIAs are introduced in detail in this section to show the work mechanism and realization process of the BIAs inspired from evolution, namely, Selfish Gene Algorithm (the level of gene evolution), Invasive Weed Algorithm (the level of population evolution), and Culture Algorithm (the level of society evolution). The first two algorithms belong to BIAs based on biological evolution, and Culture Algorithm is based on society evolution.

#### 3.3.1. Selfish Gene Algorithm

Selfish Gene Algorithm is a new member of BIAs, which is based on the selfish gene theory presented by Dawkins [42]. In the selfish gene theory, the evolution is suggested to be viewed as acting at gene level. The selection in organisms or populations is based on genes. In addition, the population is seen as a genes pool where the number of individuals and their specific identities are not of interest. So Selfish Gene Algorithm focuses on the fitness of genes rather than individuals. The main concepts in Selfish Gene Algorithm are summarized as follows [43, 44] and the pseudocode of Selfish Gene Algorithm is summarized in Algorithm 1.

*(1) Virtual Population.* The virtual population aims at modeling the gene pool concept defined by Dawkins, which is denoted by a vector:

(6) $$\begin{array}{c} p=\left(\;p_{1},p_{2},\ldots ,p_{n}\;\right), \end{array}$$

where *n* is the number of genes and *p*_*i*_ indicates the survival rate of the *i*th gene. In Selfish Gene Algorithm, an individual is represented by its genome, and the position in the genome is called locus *L*_*i*_.

*(2) Allele.* The value appearing at the locus *L*_*i*_ is defined as allele *a*_*ij*_, (*j* = 1,…, *n*_*i*_), where *n*_*i*_ is the number of gene in the genome. The success of an allele is based on the frequency with which it appears in the virtual population. 

*(3) Evolution Mechanism.* The evolution of the virtual population proceeds by an unspecified kind of sexual reproduction of its individuals. So Selfish Gene Algorithm does not rely on a crossover operator and the reproduction is performed implicitly. In Selfish Gene Algorithm, evolution means that the organism which succeeds will increase its allele's frequency at the expense of its children and on the other hand organism which fails will decrease its allele's frequency.

Selfish Gene Algorithm has been successfully tested in several problems. For example, António [44] proposed a strategy based on the hybridization of Memetic Algorithm and Selfish Gene Algorithm, to overcome the difficulties in attaining a global solution for the optimization design problem of composite structures. Wang et al. [45] exploited the selfish gene theory in the approach to improve the performance of the bivariate estimation of distribution algorithms.

#### 3.3.2. Invasive Weed Algorithm

Invasive Weed Algorithm is introduced by Mehrabian and Lucas [46], which simulates the weed invasion process. Weeds have powerful viability and survival strategies which turn them to undesirable plants in agriculture. However, these features of weeds are very useful for certain computing algorithms. There are three main mechanisms of the invasive weed used in Invasive Weed Algorithm. (1) Propagation mechanism: this mechanism means that the weeds will have different reproductive chances according to their fitness. (2) Diffusion mechanism: this mechanism means that the offspring weeds will diffuse within a space in a normal distribution mode taking the parent weeds as the axis. (3) Competitive mechanism: this mechanism means that all the weeds including the parent and offspring will be selected based on their merits when the number of weeds within a space reaches the upper limit. According to the three mechanisms above, the workflow of Invasive Weed Algorithm is summarized as follows.

**Step 1 (population initialization).**

A certain number of weeds diffuse in a *D* dimension space randomly.

**Step 2 (growth and reproduction).**

When the weeds grow to bloom, they will produce seeds according to their fitness. The fitness of the parent weed and the number of its offspring weeds are in a linear relationship as follows:

(7) $$\begin{array}{c} N_{s}=\frac{f-f_{min}}{f_{max}-f_{min}}\left(\;s_{max}-s_{min}\;\right)+s_{min}, \end{array}$$

where *N*_*s*_ is the number of offspring weeds; *f* is the fitness; *f*_max_ and *f*_min_ are the maximum and the minimum fitness, respectively; *s*_max_ and *s*_min_ are the maximum and the minimum number of the offspring weeds, respectively.

**Step 3 (spatial diffusion).**

The offspring weeds diffuse into the *D* dimension space in a normal distribution mode, and the standard deviations *σ*_*i*_ of the normal distribution in different iterations are changed according to the following rule:

(8) $$\begin{array}{c} \sigma_{i}=\frac{{\left(i_{max}-i\right)}^{n}}{{\left(i_{max}\right)}^{n}}\left(\;\sigma_{initial}-\sigma_{final}\;\right)+\sigma_{final}, \end{array}$$

where *i* is the iteration number; *i*_max_ denotes the maximum iteration number; *σ*_initial_ is the initial standard deviation; *σ*_final_ is the final standard deviation; and *n* is a nonlinear harmonic index.

**Step 4 (competitive exclusion).**

When the bearing capacity of environmental resources no longer satisfies the need of all the weeds, the vulnerable groups, namely, weeds with low fitness, will be weeded out.

**Step 5 .**

If the end condition is satisfied, output the optimal solution. Otherwise go to Step 2.

There are some disadvantages in Invasive Weed Algorithm. For example, the parameter sensitivity is high, there is no information exchange among the individuals, and the searching deep is less than other algorithms such as Particle Swarm Optimization Algorithm and Expectation Maximization Algorithm. Now, some improvements have been proposed in the standard Invasive Weed Algorithm, and most of them utilize the advantages of other algorithms to develop a hybrid Invasive Weed Algorithm [47, 48].

#### 3.3.3. Culture Algorithm

The evolution progress of human society is complex, which is different from the natural selection of any other living creatures. The evolution of human society is based on the heritage of knowledge and transmission of culture. Culture can make the population evolve and adapt to the environment in a certain speed higher than that of the general biological evolution relying solely on gene inheritance. Culture Algorithm [49] is proposed to simulate the evolution of human society. In Culture Algorithm, the a priori knowledge in one field and the knowledge obtained from the evolution can be used to direct the searching process. Culture Algorithm adopts a double evolutionary mechanism, to simulate the culture evolution process of human society. A reliability space is established to extract various information in the evolutionary process based on the traditional swarm evolutionary algorithms. And the information is stored in the form of knowledge, which is used to lead the evolutionary process.

There are three main parts in Culture Algorithm [5, 50]. (1) Population space: the population space is used to realize any evolutionary algorithm based on population, where individuals are evaluated and the selection, crossover, and mutation evolutionary operations are conducted. In addition, the excellent individuals are provided to the reliability space as samples. (2) Reliability space: the reliability space receives samples from the population space by the acceptance function and selects the information in samples by the update function, which is abstracted, described, and stored in the form of knowledge. Finally, various knowledge acts on the population space by the influence function, to accelerate the convergence of the evolution and improve the environmental adaptability of the algorithm. (3) Communication channels: the communication channels include the acceptance function, the update function, and the influence function. The population space and the reliability space are two independent evolution processes. The acceptance function and the influence function provide channels for the upper knowledge model and the lower evolutionary process, so the two functions are also called interface functions. The basic structure of Culture Algorithm can be seen in [5].

Culture Algorithms offer power alternation for search problems, which can also provide an understanding of cultural phenomena and underlying technology utilized by human species. Culture Algorithm can be used to deal with pattern recognition, multirobot coordination, fault classification, and so on [50–52].

A brief summary of the bioinspired intelligent algorithms, presented in the subsections above, is illustrated in Table 1, where the main advantages and applications of these algorithms are given out. And the related literatures are also listed.

## 4. Applications Overview of BIAs for Mobile Robot Control

As introduced in Section 3, BIAs have been used widely in lots of fields. To introduce BIAs clearly, we focus exclusively on the application field in mobile robot control in this section, which is one of the most important application fields of BIAs. The main applications for mobile robot control based on BIAs summarized here are based on what the authors are most aware of. Although not comprehensive, the applications cited here can demonstrate some of the key features of BIAs. To reduce the repetition and introduce more BIAs, the BIAs introduced in this section are different from those in Section 3.

Mobile robot, as an important robotic research branch, develops very quickly, which can be used to complete various tasks that do not suit humans, such as when the working environment is dangerous and poisonous, the task is full of sameness and dull repetition, and the exploration task is in the deep sea or outer space [61–63]. The main problems that must be addressed in mobile robot control include path planning, simultaneous localization and mapping, and cooperative control of multirobots, which will be introduced in detail as follows.

### 4.1. Path Planning

Path planning is one of the basic problems in the mobile robot control field, which is very important to realize the intelligence and autonomy of mobile robots. The goal of path planning is to find an optimal or suboptimal path from the starting position to the target position [64, 65]. Various methods have been used to deal with path planning problems, such as the potential field methods and the fuzzy control methods. These methods have achieved certain success. However, there are still some problems needed to be further studied, including path planning in unknown and dynamic environments and the local minimum problem of most optimization algorithms. Recently, some BIAs have been proposed in solving the problems in path planning of mobile robots. The detailed information of typical BIAs in robot path planning is described in the sequel.

**Figure 4:**
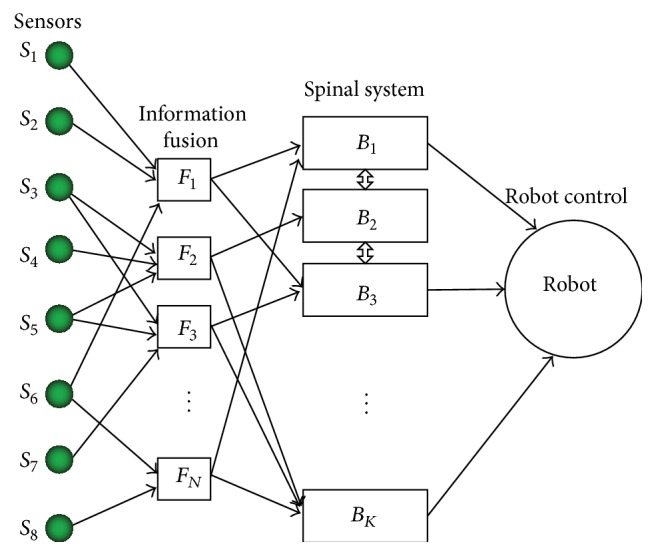
Mapping among sensors, fusion units, and spinal system [66].

Siddique and Amavasai [66] proposed a behavior based controller inspired by the concept of spinal fields found in frogs and rats. The core idea of this bioinspired behavior-based controller is that the robot controller consists of a collection of spinal fields which are responsible for generating behaviors depending on their activation levels. The collection of spinal fields is denoted by {*B*_1_, *B*_2_, *B*_3_,…, *B*_*K*_}. The set of sensor fusion units is denoted by {*F*_1_, *F*_2_,…, *F*_*N*_}. The mapping among sensors, fusion units, and spinal fields is shown in Figure 4. In their study, four basic behaviors are chosen, namely, {go  forward}, {turn  left},   {turn  right}, and  {go  back}. The four behaviors are mapped into the corresponding robot wheel speeds {*V*_*L*_, *V*_*R*_}. Then a function of spinal fields to realize the behavior is expressed as {*V*_*L*_, *V*_*R*_} = *f*(*B*_1_, *B*_2_, *B*_3_, *B*_4_). Finally, four experiments are carried out with Khepera robot, and the results show that their developed bioinspired behavior-based controller is simple but robust.

Yang and Meng [67] used a bioinspired neural network to realize the dynamic collision-free trajectory generation for mobile robot in a nonstationary environment. This bioinspired neural network is based on a shunting model, which is obtained from a computational membrane model for a patch of membrane in a biological neural system (proposed by Hodgkin and Huxley in 1952 [23]). The core idea of this bioinspired neural network based robot trajectory generation method is that the neural network is topologically organized, where the dynamics of each neuron is characterized by a shunting equation:

(9) $$\begin{array}{c} \frac{dx_{i}}{dt}=-Ax_{i}+\left(\;B-x_{i}\;\right)S_{i}^{+}-\left(\;D+x_{i}\;\right)S_{i}^{-}, \end{array}$$

where *x*_*i*_ is the neural activity (membrane potential) of the *i*th neuron; *A*, *B*, and *D* are nonnegative constants, representing the passive decay rate and the upper and lower bounds of the neural activity, respectively; and *S*_*i*_^+^ and *S*_*i*_^−^ are the excitatory and inhibitory inputs to the neuron. The excitatory input *S*_*i*_^+^ results from the target and the lateral connections from its neighboring neurons, while the inhibitory input *S*_*i*_^−^ results from obstacles only. Thus the differential equation for the *i*th neuron is given by

**Figure 5:**
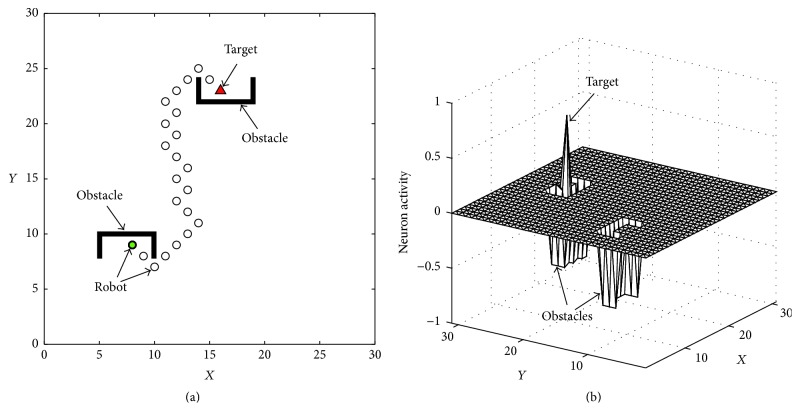
Real-time path planning of robot by the bioinspired neural network: (a) the generated path of the robot; (b) the neural activity of the bioinspired neural network.

(10) dxidt=−Axi+B−xiIi++∑j=1qwijxj+−D+xiIi−,

where *q* is the number of neural connections of the *i*th neuron to its neighboring neurons within a receptive field; *w*_*ij*_ is the lateral connection weight from the *i*th neuron to the *j*th neuron; function [*ζ*]^+^ is defined as [*ζ*]^+^ = max{*ζ*, 0}, and function [*ζ*]^−^ is defined as [*ζ*]^−^ = max{−*ζ*, 0}. *I*_*i*_ is the external input to the *i*th neuron, which is defined as

(11) $$\begin{array}{c} I_{i}=\left\{\begin{array}{cc} E, & if\;\;there\;\;is\;\;a\;\;target\\ -E, & if\;\;there\;\;is\;\;an\;\;obstacle\\ 0, & otherwise, \end{array}\right. \end{array}$$

where *E* is a positive constant and *E* ≫ *B*. The dynamic activity landscape of the topologically organized neural network is used to determine the next robot location:

(12) $$\begin{array}{c} p_{n}⟸x_{p_{n}}=max\left\{\;x_{j},\;j=1,2,\ldots ,k\;\right\}, \end{array}$$

where *x*_*j*_, *j* = 1,2,…, *k*, is the activity of all the neighboring neurons of the present neuron (the present location of the robot); *p*_*n*_ is the location of the neuron with the maximum activity in these neurons (the next possible locations of the robot). One of the path planning results in a U-shaped environment based on this bioinspired neural network is shown in Figure 5, where the generated path is shown in Figure 5(a), while the neural activity landscape in 3D is shown in Figure 5(b).

### 4.2. Simultaneous Localization and Mapping

The solution to the simultaneous localization and mapping (SLAM) problem is a key issue in the field of mobile robot control, which is regarded as a “Holy Grail” of autonomous mobile robot [68]. The core task of SLAM is that a robot explores in an unknown environment to learn the environment (map) by utilizing the sensors onboard, while simultaneously using that map to locate within the environment. A number of approaches have been proposed to address the SLAM problem, and the most typical and widely used SLAM algorithm is EKF-based SLAM at present. However, the SLAM problems have not been resolved efficiently. For example, the accuracy of the system noise and the observation noise model will decide the final accuracy of the EKF-based SLAM algorithms. Recently, more and more attention has been focused on emulating the biological system thought to be responsible for mapping and navigation in animals, which are introduced as follows.

Milford and Wyeth [69] investigated the persistent navigation and mapping problem of an autonomous robot, and a biologically inspired SLAM system based on models of mapping in the rodent hippocampus (RatSLAM) was presented. The proposed RatSLAM consists of three components: a set of local view cells, a network of pose cells, and an experience map (see [69] for details). The pose cells are a three-dimensional continuous attractor network (CAN). For each pose cell, local excitation and inhibition are achieved through a three-dimensional Gaussian distribution of weighted connections. The distribution *ɛ* is given by

(13) $$\begin{array}{c} \varepsilon_{a,b,c}=e^{-\left(a^{2}+b^{2}\right)/k_{p}^{exc}}e^{-c^{2}/k_{d}^{exc}}-e^{-\left(a^{2}+b^{2}\right)/k_{p}^{inh}}e^{-c^{2}/k_{d}^{inh}}, \end{array}$$

where *k*_*p*_ and *k*_*d*_ are variant constants for place and direction, respectively, and *a*, *b*, and *c* represent the distances between units in *x*′, *y*′, and *θ*′ coordinates, respectively. The local view cells are an array of rate-coded units used to represent what the robot is seeing. The connections between local view cell *V*_*i*_ and pose cell *P*_*x*′,*y*′,*θ*′_ are stored in a matrix *β*, which are calculated by

(14) $$\begin{array}{c} \beta_{i,x^{\prime },y^{\prime },\theta^{\prime }}^{t+1}=max\left(\;\beta_{i,x^{\prime },y^{\prime },\theta^{\prime }}^{t},\lambda V_{i}P_{x^{\prime },y^{\prime },\theta^{\prime }}\;\right), \end{array}$$

where *λ* is the learning rate. The experience map is a semimetric topological map containing representations of places (called experiences) and links among experiences describing the transitions among these places. RatSLAM performs SLAM continuously while interacting with global and local navigation systems and a task selection module that selects tasks among exploration, delivery, and recharging modes. The real robot experiments are conducted, where the workplaces are floors in two different buildings at The University of Queensland in Brisbane, Australia. The results demonstrate that RatSLAM can serve as a reliable mapping resource for an autonomous system in multiple environments.

Barrera and Weitzenfeld [24] presented a robot architecture with spatial cognition and navigation capabilities that captures some properties of rat brain structures involved in learning and memory. The detailed biological framework of their proposed model can be seen in their paper, which includes four main modules: path integration module, landmarks processing module, place representation module, and learning module. The basic procedures of this model are summarized as follows: (1) the sensory information including kinesthetic information, visual information, and affordances information is input into the posterior parietal cortex (PPC) module, which is suggested as part of a neural network mediating path integration; (2) the hippocampus receives kinesthetic and visual information from the retrosplenial cortex and the entorhinal cortex existing in PPC module, respectively; (3) the internal state is combined with incentives, which are under control of the lateral hypothalamus; (4) all the process results (including the affordances perceptual schema, the landmark perceptual schema, and the learning results) are integrated into the place representation module comprising a place cell layer and a world graph layer, to obtain the decision-making basis for the action selection module, which will determine the robotic motor outputs. From a robotics perspective, this work can be placed in the gap between mapping and map exploitation currently existent in the SLAM literature.

### 4.3. Cooperation Control

Multirobot systems have been a subject of much research since the 1970s for various tasks. The coordination of multirobot systems has recently attracted more and more attention, because a mobile robot team can accomplish a task rapidly and efficiently [6, 70]. A lot of work has been done on cooperation in multirobot systems, and the main issues in this field include hunting, scheduling, and foraging. In this paper, some literature related to these issues solved by BIAs is overviewed.

Tan et al. [71] proposed a multirobot cooperative control algorithm for environment exploration based on immune network model of B-T-cell. In their algorithm, the environment which will be detected is considered as an antigen. The robot is considered as a B-cell. The robotic behavior strategies are considered as antibodies produced by B-cells. The control parameters of the algorithm are equivalent to the regulatory effects of T-cells. The dynamic equation for the antibody levels of incentives and concentration is as follows:

(15) Θit=Θit−1+ΔΘit−1·θit−1,ΔΘit−1=α∑j=1Nmijθjt−1N−∑k=1Nrkiθkt−1N−cit−1−ki+βgi,

where Θ_*i*_(*t*) is the incentive level of the *i*th antibody; *θ*_*i*_(*t*) is the concentration of the *i*th antibody; *N* is the number of the antibodies; *m*_*ij*_ represents the affinity coefficients between the *i*th and *j*th antibody; *r*_*ki*_ represents the rejection coefficient between the *k*th and *i*th antibody; *α* represents the interaction rate between the *i*th antibody and other antibodies; *β* represents the interaction rate between the *i*th antibody and the antigen; *c*_*i*_(*t*) represents the concentration of T-cell which regulates the antibody concentration of B-cell; *k*_*i*_ represents the natural mortality rate of the *i*th antibody; and *g*_*i*_ represents the matching rate between the *i*th antibody and the antigen. Each robot detects the environment according to the matching rate between the antibody and the antigen, till the exploration in the environment is completed.

Yang and Zhuang [72] presented an improved Ant Colony Optimization Algorithm for solving mobile agent (robot) routing problem. The ants cooperate using an indirect form of communication mediated by pheromone trails of scent and find the best solution to their tasks guided by both information (exploitation) which has been acquired and search (exploration) of the new route. When the ant *k* travels, the probability that the ant *k* chooses the next host computer to be visited is

(16) pkr,s=τr,sps/dr,s·tsβ∑u∈Jirτr,upu/dr,u·tuβ,s∈Jkr,0,otherwise,

where *p*_*k*_(*r*, *s*) is the probability that the ant *k* chooses the *s*th host computer as the next destination; *r* is the current host computer which the ant is at; *d*(*i*, *j*) is the time that a mobile agent needs from the *i*th to the *j*th host computer; *τ*(*i*, *j*) is the pheromone trail on the route between the *i*th and *j*th host computer; *p*_*i*_ is the probability that the mobile agent completes the task in the *i*th host computer, and then the time delay in this host computer is denoted as *t*_*i*_; *J*_*i*_(*r*) is the set of the host computers that remain to be visited by the ant *i* positioned in the *r*th host computer; *β*  (0 ≤ *β* ≤ 1) is a parameter to control the tradeoff between visibility (constructive heuristic) and pheromone trail concentration. Then the more the pheromone trails on the route that the ants choose, the shorter the delay time and the higher the probability that the ants will choose that route. The pheromone trails on the route can evaporate as time goes on, and also the pheromone trails in the route can be updated after all ants complete their tours, respectively, and return to the initial host computer. The algorithm has been successfully integrated into a simulated humanoid robot system which won the fourth place of RoboCup 2008 World Competition.

### 4.4. Other Applications

Lots of studies have been done on BIAs in mobile robot control besides those introduced above, such as machine vision, robotic exploration, and olfactory tracking.

**Figure 6:**
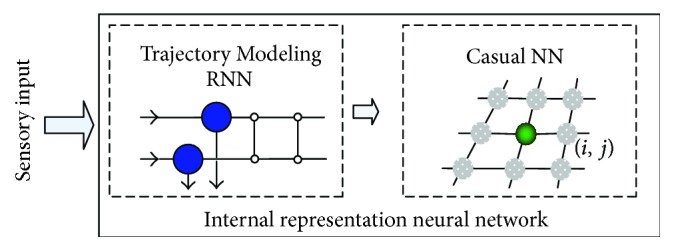
The sketch map of the internal representation neural network (IRNN) [73].

Villacorta-Atienza et al. [73] proposed an internal representation neural network (IRNN), which can create compact internal representations (CIRs) of dynamic situations, describing the behavior of a mobile agent in an environment with moving obstacles. Emergence of a CIR in IRNN can be viewed as a result of virtual exploration of the environment. The general architecture of this IRNN is shown in Figure 6, which consists of two coupled subnetworks: Trajectory Modeling RNN (TM-RNN) and Causal Neural Network (CNN). The output of TM-RNN is time independent, and hence it just maps the immobile objects into CNN whose dynamics model the process of virtual exploration

(17) $$\begin{array}{c} {\dot{r}}_{ij}=d\cdot \Delta r_{ij}-r_{ij}\cdot p_{ij}, \end{array}$$

where *r*_*ij*_ is the neuron state variable, representing the concentration of virtual agents at the cell (*i*, *j*); the time derivative is taken with respect to the mental (inner) time *τ*; Δ*r*_*ij*_ = (*r*_*i*+1,*j*_ + *r*_*i*−1,*j*_ + *r*_*i*,*j*+1_ + *r*_*i*,*j*−1_ − 4*r*_*ij*_) denotes the discrete Laplace operator describing the local (nearest neighbor) interneuronal coupling, whose strength is controlled by *d* (a constant number); and *p*_*ij*_ accounts for the target; if (*i*, *j*) is occupied by a target, *p*_*ij*_ = 1; otherwise *p*_*ij*_ = 0. In their work, the effectiveness of IRNN is proved by some tests in different simulated environments, including the environment with a single moving obstacle and the realistic environments.

Sturzl and Moller [74] presented an insect-inspired approach to orientation estimation for panoramic images. The relative orientation to a reference image *I* ^*a*^ can be estimated by simply calculating the image rotation that minimizes the difference to the current image *I* ^*b*^; that is,

(18) ϕ^ab=arg minϕ∑iωiabϕ−1·∑iωiabϕIiaϕ−Iib2,

where *I* ^*a*^(*ϕ*) denotes image *I* ^*a*^ rotated by angle *ϕ* around an axis and the weights *ω*_*i*_^*ab*^(*ϕ*) are calculated using *ω*_*i*_^*ab*^(*ϕ*) = (var_*i*_^*a*^(*ϕ*) + var_*i*_^*b*^(0))^−1^, where var_*i*_^−1^ is supposed to give information about the quality of the pixel for rotation estimation. For example, it should be low if the pixel belongs to an image part showing close objects. In their minimalistic approach, the varivalues are computed from pixel differences of images recorded at nearby position with the same orientation.

Martinez et al. [56] presented a biomimetic robot for tracking specific odors in turbulent plumes, where an olfactory robot was constructed, which consisted of a Koala robot with an onboard computer and two electronic noses (E-noses) placed on both sides of the robot. And a simple biologically inspired strategy was proposed, where the robot was navigated with two spatially separated sensor arrays. Let *C*_*l*_(*t*) and *C*_*r*_(*t*) be the odor concentrations estimated at time *t* by the sensors at the left and right, respectively. The concentration difference *C*_*l*_(*t*) − *C*_*r*_(*t*) is noted as Δ*C*(*t*). When only the side hit by an odor patch is known, the turning speed of the robot *ω* would be

(19) $$\begin{array}{c} \omega \left(\;t\;\right)=\Omega \left(\;t\;\right)\cdot \underset{}{sgn}\left(\;\Delta C\left(\;t\;\right)\;\right), \end{array}$$

where *Ω*(*t*) is an adaptive parameter for the robotic turning speed. The proposed bioinspired method is well suited for building dedicated circuits and onboard implementation on real robots. Similarly, a bioinspired autonomous swimming robot is designed to study the goal-directed locomotion, which is inspired by the lamprey. The robotic lamprey is controlled by a simulated central pattern generator network that generates the species-specific locomotor movements (see [75] for details).

## 5. Future Directions

Bioinspired intelligent computing is a relatively novel interdisciplinary field of research, and there are many theoretical problems needed to be solved. The most important and widely studied theoretical problems are convergence problem, effectiveness problem, and evaluation standard problem. Because most of the BIAs are based on probability searching, it is difficult to rigorously prove the convergence and effectiveness of BIAs in mathematics. Currently, many theoretical research results are aiming at one specific algorithm to carry out the research work. One of the future works is to integrate some related algorithms to a common theory framework for the proof of convergence and effectiveness. In addition, the evaluation of BIAs should be conducted in a specific aspect for a specific task, such as the computing speed or the correctness of the solution, because it is difficult to find a BIA, which is suitable for all the problems. When we design an algorithm, the practical principle should be followed, and the algorithm with unnecessary complex should not be selected. Other research branches in the theory study of BIAs include the selection problem of initial parameters and the problem of convergence speed. We believe that lots of novel BIAs will be developed constantly with the deepening of biological research.

In the future, BIAs will play an important role in more and more fields. In the application field for mobile robot control based on BIAs, the main future directions include bioinspired sensors, cognitive model, and bioinspired robots. In the robotic sensors, the development of the traditional sensors meets a big problem; that is, it becomes more and more difficult to improve the precision of the sensors, and the production cost of the sensors is higher and higher. However, as we know, lots of organisms do not have any high-precision sensors, while they can achieve perfect sense function. So the bioinspired sensors may be a good solution for the robotic sensors. In the cognitive model, currently many research results have been obtained based on the working mechanism of the brains of rats and human beings. Another important issue which should be of concern is the performance of BIAs in real applications of mobile robots. In the near future, the robot with high intelligence, self-learning, and self-perception will become the mainstream in the mobile robot field.

## 6. Conclusions

In this survey we have analyzed the main features of BIAs based on our work and the overview of the literature and given out a classification of BIAs from the biomimetic mechanism. According to the classification, we have surveyed the realization process of each category of BIAs by some concrete algorithms selected carefully. Furthermore, we have surveyed the applications of BIAs focused on the mobile robot control field. Finally, we have provided some possible directions for future study. Due to the large and growing literature in this area, many interesting results have not been included in an attempt to capture some of the key areas in this field.

It is clear that BIAs are going to be one of the hottest research points in the computation intelligent field, including theory and application research. And BIAs will play an important role in mobile robot control, which will be a good solution to improve the intelligence and autonomy of mobile robot and can solve the development bottleneck of traditional technologies, such as the balance between precision and cost. Currently, many basic problems of mobile robot control based on BIAs have been explored and the results are exciting to demonstrate the potential of BIAs. However, most of the results available are only conducted by simulation; additional efforts are needed to develop some more efficient BIAs and transit these results to real applications in mobile robot control.

## Figures and Tables

**Algorithm 1:**
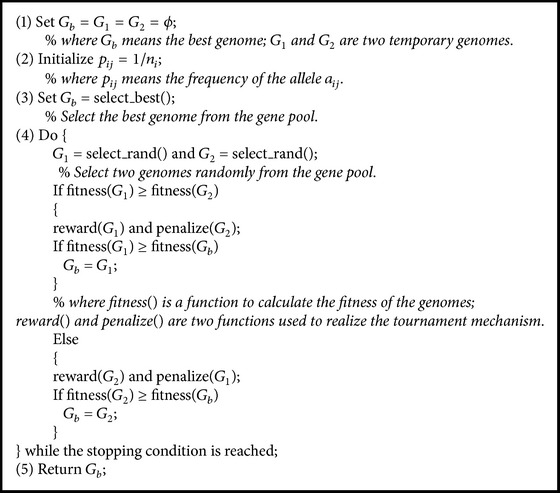


**Table 1:** A brief summary of the BIAs introduced in this paper.

Category	Name	Advantages	Applications	References
Inspired from organism behavior	Bacterial Foraging Algorithm	Parameter insensitivity; strong robustness; easy implementation	Image segmentation; robot path planning; optimum scheduling; optimal power flow	[11–15, 53]
	Monkey Climbing Algorithm	A few parameters to adjust; low calculation cost; fast convergence rate	Optimal sensor placement; feature selection and extraction; numerical optimization	[16–19, 54]

Inspired from organism structure	DNA Computing	High parallelism; massive storage ability; low energy consumption	Information security; robotic control; task assignment problem; clustering problem	[20, 25–28, 55]
	Membrane Computing	Inherent parallelism; distributed feature; nondeterminism	Numerical optimization; broadcasting problem; computer graphics; traveling salesman problem	[21, 29–34]
	Artificial Immune System	Noise patience; learning without teacher; self-organization and identity	Community detection; anomaly detection; fault diagnosis; web page classification	[5, 22, 35–41]

Inspired from evolution	Selfish Gene Algorithm	High convergent reliability and convergent velocity	Optimization design problem; traveling salesman problem; scheduling problem	[42–45, 57]
	Invasive Weed Algorithm	Easy to understand; good adaptability; strong robustness	Image clustering problem; parameter estimation problem; numerical optimization	[46–48, 58, 59]
	Culture Algorithm	With a double evolution structure; high search efficiency; with a certain universality	Pattern recognition; multirobot coordination; fault classification; engineering design problem	[5, 49–52, 60]
